# Influenza A virus activates cellular Tropomyosin receptor kinase A (TrkA) signaling to promote viral replication and lung inflammation

**DOI:** 10.1371/journal.ppat.1010874

**Published:** 2022-09-19

**Authors:** Vikram Verma, Mythili Dileepan, Qinfeng Huang, Thu Phan, Wei-Shou Hu, Hinh Ly, Yuying Liang

**Affiliations:** 1 Department of Veterinary and Biomedical Sciences, College of Veterinary Medicine, University of Minnesota, St Paul, Minnesota, United States of America; 2 Department of Chemical Engineering and Material Sciences, College of Science and Engineering, University of Minnesota, Minneapolis, Minnesota, United States of America; Johns Hopkins Bloomberg School of Public Health, UNITED STATES

## Abstract

Influenza A virus (IAV) infection causes acute respiratory disease with potential severe and deadly complications. Viral pathogenesis is not only due to the direct cytopathic effect of viral infections but also to the exacerbated host inflammatory responses. Influenza viral infection can activate various host signaling pathways that function to activate or inhibit viral replication. Our previous studies have shown that a receptor tyrosine kinase TrkA plays an important role in the replication of influenza viruses *in vitro*, but its biological roles and functional mechanisms in influenza viral infection have not been characterized. Here we show that IAV infection strongly activates TrkA *in vitro* and *in vivo*. Using a chemical-genetic approach to specifically control TrkA kinase activity through a small molecule compound 1NMPP1 in a TrkA knock-in (TrkA KI) mouse model, we show that 1NMPP1-mediated TrkA inhibition completely protected mice from a lethal IAV infection by significantly reducing viral loads and lung inflammation. Using primary lung cells isolated from the TrkA KI mice, we show that specific TrkA inhibition reduced IAV viral RNA synthesis in airway epithelial cells (AECs) but not in alveolar macrophages (AMs). Transcriptomic analysis confirmed the cell-type-specific role of TrkA in viral RNA synthesis, and identified distinct gene expression patterns under the TrkA regulation in IAV-infected AECs and AMs. Among the TrkA-activated targets are various proinflammatory cytokines and chemokines such as IL6, IL-1β, IFNs, CCL-5, and CXCL9, supporting the role of TrkA in mediating lung inflammation. Indeed, while TrkA inhibitor 1NMPP1 administered after the peak of IAV replication had no effect on viral load, it was able to decrease lung inflammation and provided partial protection in mice. Taken together, our results have demonstrated for the first time an important biological role of TrkA signaling in IAV infection, identified its cell-type-specific contribution to viral replication, and revealed its functional mechanism in virus-induced lung inflammation. This study suggests TrkA as a novel host target for therapeutic development against influenza viral disease.

## Author summary

Despite the annual vaccination program and several antiviral options, influenza viruses continue to cause annual epidemics, occasional zoonotic infections and pandemics in humans. Effective antiviral therapeutics are needed to combat severe influenza respiratory disease, which is caused by both the direct viral cytopathic effect and the exacerbated host inflammatory responses. In this study, we show that a host receptor tyrosine kinase TrkA signaling is functionally important for promoting both influenza viral replication and virus-induced lung inflammation. Using a unique mouse model and its derived primary lung cells, in which the TrkA kinase activity can be specifically controlled by a small compound inhibitor, we demonstrated the important role of TrkA in the influenza A viral infection in animals and revealed its cell-type-specific functional mechanisms in promoting viral replication and lung inflammation. Our study suggests TrkA as a potential novel host target for developing effective therapeutics against influenza viral diseases.

## Introduction

Influenza A virus (IAV) infection leads to acute lung disease with potential severe and deadly complications, and poses a significant public health concern by causing annual epidemics and occasional zoonotic infections and pandemics [1]. Current vaccination programs and the existing antivirals are insufficient to provide effective prevention or treatment against influenza infections, partly due to the constantly evolving nature of IAVs resulting in the emergence of vaccine escape and drug-resistant viral variants [2]. Targeting host factors that are important for viral replication and disease development represents a viable alternative approach, but it requires an in-depth understanding of the functional mechanisms of these host factors in mediating viral replication and pathogenesis.

### TrkA receptor tyrosine kinase signaling plays an important role in the influenza viral replication

Multiple cellular signaling pathways have been shown to play important roles in the IAV life cycle. The Nuclear Factor Kappa B (NF-kappaB) signaling facilitates IAV replication by promoting the replication of viral RNAs [3–5]. The epidermal growth factor receptor (EGFR) is activated by IAV infection and facilitates efficient viral entry [6]. The phospholipase C gamma 1 (PLC-γ1) signaling is critical for cellular uptake of H1N1 but not H3N2 virus [7]. Inhibition of the phosphoinositide 3-kinase (PI3K) and Raf/MEK/ERK was shown to reduce production of viral particles [8,9]. Our previous studies suggested that a receptor tyrosine kinase (RTK) known as tropomyosin receptor kinase A (TrkA) is important for efficient influenza viral replication [10,11]. We have shown that multiple TrkA inhibitors strongly block the production of various influenza A and B virus strains, including A/WSN (H1N1), A/PR8 (H1N1), A/x31 (H3N2), and B/Victoria, by > 2 logs in human lung epithelial A549 cells and Madin-Darby canine kidney (MDCK) cells [10,11], and that inhibition occurs at multiple steps of the viral life cycle–viral RNA synthesis, nuclear export of viral ribonucleoprotein complex (vRNPs), and viral budding and release [11]. The role of TrkA in promoting the influenza viral replication is also demonstrated in shRNA-mediated knockdown and over-expression experiments [11]. TrkA-shRNA knockdown in human lung epithelial A549 cells significantly reduced IAV titer, while viral production was much higher in SN56-TrkA, which are TrkA-overexpressing neuroblastoma cells, than in control SN56 cells. Nevertheless, the functional role(s) of TrkA in IAV replication and pathogenesis in the lungs have not been characterized.

### Pathophysiological role of TrkA signaling in the lung

TrkA, also known as neurotrophic tyrosine kinase receptor type 1 (NRTK1), is a member of the Trk family of receptor tyrosine kinases and the high-affinity receptor for nerver growth factor (NGF) [12,13]. Ligand binding induces the dimerization and autophosphorylation of Trk receptors at the Tyr residues [14–17], which then recruit adaptor proteins that activate or link to the downstream PI3K-Akt, Ras-ERK-MAPK, and PLC signaling pathways [12,13,18,19]. NGF-activated TrkA signaling cascades have been shown to promote neuronal cell growth or differentiation [13,19–21]. Although the NGF-TrkA signaling has largely been characterized in neurons, TrkA is also expressed in a wide variety of non-neural tissues including human lung [22–24]. TrkA expression is detected in eosinophils, bronchial and alveolar epithelial cells, smooth muscle cells, and vascular endothelial cells in the lungs [24]. However, the physiological roles of NGF-TrkA signaling in the lungs are unclear. Evidence from clinical observation and animal models of allergen-induced airway inflammation (AAI) supports an important role for NGF/TrkA signaling in allergic asthma [25–29]. Using a chemical-genetic approach to specifically control TrkA kinase activity *in vitro* and *in vivo*, we have demonstrated that TrkA activation is important for AAI by promoting eosinophil recruitment through a distinct eotaxin-mediated TrkA-dependent signaling pathway [30].

### A chemical-genetic approach to specifically control TrkA kinase activity

Gene knockout of NGF or TrkA in mice caused lethal phenotype due to defective neuron development [31,32]. Most commonly used TrkA inhibitors, like other tyrosine kinase inhibitors, have limited drug specificity and selectivity [33,34]. To overcome this challenge, a chemical-genetic strategy has been developed to conveniently and specifically control kinase activity through small molecule compounds *in vitro* and *in vivo* [35]. A phenylalanine (F)-to-alanine (A) substitution within the kinase domain of TrkA (F592A) does not affect its ATP-binding activity but renders it sensitive to specific inhibition by a small-molecule PP1 derivative 1NMPP1 [36]. Chen et al. used the gene-targeting approach to create homozygous TrkA^F592A^ knockin (TrkA KI) mice, which are viable and fertile with no obvious behavioral abnormality, and their TrkA signaling can be specifically, potently, and reversibly inhibited by 1NMPP1 [36].

In this study, we show that IAV infection activates the TrkA signaling *in vitro* and *in vivo*. Using the TrkA KI mouse model, we demonstrate that TrkA plays specific and important roles in both IAV replication and virus-induced lung inflammation. Through transcriptomic analysis of primary alveolar epithelial cells (AECs) and alveolar macrophages (AMs) infected with IAV in the presence or absence of 1NMPP1, we show that TrkA activation is important for IAV RNA replication in AECs but not in AMs, and that TrkA activation upregulates proinflammatory gene expression in both cell types. Our study reveals novel cell-type-specific functional mechanisms of the TrkA signaling in promoting the IAV replication and virus-induced lung inflammation, suggesting TrkA as a novel host target for anti-influenza therapeutics.

## Results

### IAV infection activates the TrkA signaling *in vitro* and *in vivo*

**Fig 1:**
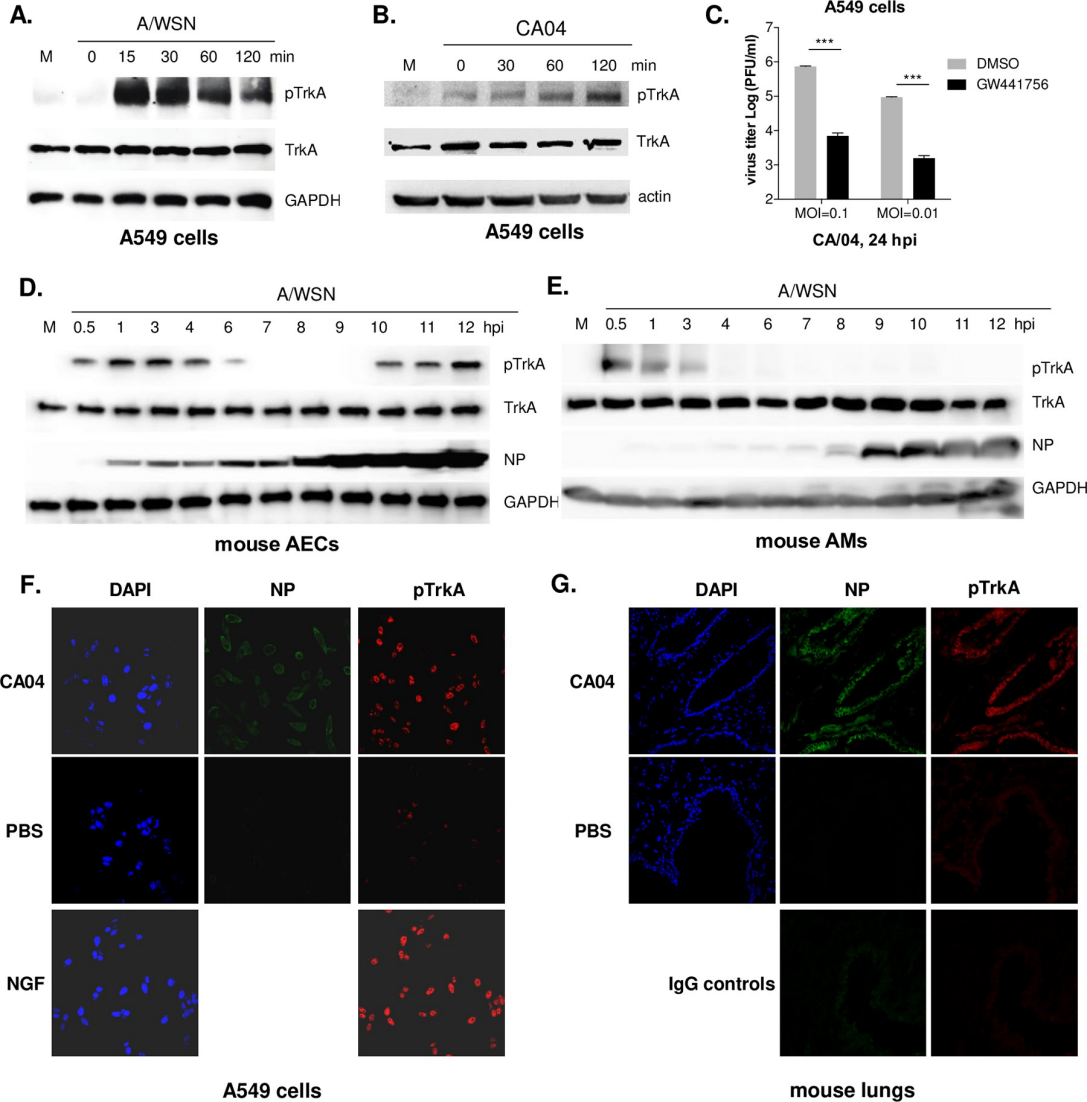
Influenza viral infection activated TrkA *in vitro* and *in vivo*. Human airway epithelial A549 cells were infected with A/WSN at MOI of 10 (**A**) or with 2009 H1N1pdm CA/04 at MOI of 1 (**B**). Primary mouse airway epithelial cells (AECs) (**D**) or primary mouse alveolar macrophages (AMs) (**E**) were infected with A/WSN at MOI of 10. Cell lysates at different times post infection were analyzed by Western blotting using antibodies against total and phosphorylated TrkA (pTrkA), viral NP, GAPDH or actin. **(C)** TrkA inhibition reduces A/CA04 replication in human airway epithelial cells. A549 cells were infected with CA/04 at MOI of 0.1 or 0.01, in the presence of DMSO or TrkA inhibitor GW441756 at 10 μM. Viral titers at 24 hpi were quantified by plaque assay. Statistical analysis of viral titer and BALF cell count was conducted by Student’s t-test. ***, p<0.001. (**F**) A549 cells were mock infected (PBS) or infected with 2009 H1N1pdm CA/04 at MOI of 10 for 1 h or treated with NGF for 30 min. Fixed cells were analyzed by IFA with DAPI, anti-NP (FITC), and anti-pTrkA (Rhodamin) antibodies. (**G**) BL6 mice were mock infected (PBS) or infected (i.n.) with A/CA04 for 3 days. Lung sections were stained with FITC-labeled anti-NP and Rhodamin-labeled anti-pTrkA antibodies, or IgG controls. IFA images were taken under a confocal microscope.

To determine whether IAV infection activates the TrkA signaling, we infected human airway epithelial cells A549 with A/WSN strain at the multiplicity of infection (MOI) of 10 and prepared cell lysates from 0 to 120 min post-infection for Western blotting with antibodies against total TrkA, phosphorylated TrkA (pTrkA), or GAPDH. While total TrkA and GAPDH remained similar, strong pTrkA was detected as early as 15 min post-infection (**Fig 1A**), demonstrating that IAV infection activates TrkA early in the infection. Similarly, pTrkA was detected early in the infection of A549 cells with 2009 H1N1 pandemic (H1N1pdm) strain A/CA04 at MOI of 1 (**Fig 1B**). Inhibition of TrkA signaling by the TrkA inhibitor GW441756 was shown to significantly reduce viral production by ~ 2 logs in A549 cells infected with A/CA04 at both MOI of 0.1 and 0.01 (**Fig 1C**), consistent with our previous findings for A/WSN, A/PR8, A/x31 (H3N2), or B/Victoria infection of A549 cells [10,11].

We also examined the TrkA activation in primary airway epithelial cells (AECs) and alveolar macrophages (AMs), the main target cells of influenza virus *in vivo* [37,38]. Primary AECs and AMs were isolated from BL6 mice and infected with A/WSN at MOI of 10. At different times post-infection, cell lysates were prepared and analyzed by Western blotting with antibodies against total TrkA, pTrkA, NP, and GAPDH, respectively (**Fig 1D and 1E**). In both virus-infected AECs and AMs, the level of viral NP protein increased sharply at 8 to 9 hpi, demonstrating successful viral RNA transcription and protein expression. Total TrkA was readily detected in both AECs and AMs, consistent with the previous reports that TrkA is expressed in various lung cell types including epithelial cells and macrophages [23,24]. In virus-infected AECs, the level of pTrkA increased from 0.5 to 6 hpi, decreased from 6 to 9 hpi, and re-appeared at 10 hpi that was likely a result of the second round of viral infection (**Fig 1D**). In virus-infected AMs, pTrkA was detected early in the infection from 0.5 to 3 hpi, but did not re-appear at later time points (**Fig 1E**), possibly due to the fact that many IAVs, including the A/WSN strain, caused abortive infections in AMs and did not initiate the secondary round of infection [39–42]. Taken together, our data suggest that IAV infection activates TrkA signaling early after infection in both AECs and AMs.

We next conducted immunofluorescence assay (IFA) to validate the TrkA activation in cultured cells and in mouse lungs after the infection of 2009 H1N1 pandemic (H1N1pdm) strain A/CA04. A549 cells were mock infected with PBS, infected with A/CA04, or treated with the high-affinity TrkA ligand NGF. IFA was conducted with antibodies against influenza NP and pTrkA. In contrast to the low level of pTrkA in PBS-treated cells, high levels of pTrkA signals were detected after NGF treatment and after A/CA04 infection (**Fig 1F**). To detect the TrkA activation in IAV-infected lungs, we infected BL6 mice with A/CA04 intranasally and conducted IFA on lung sections at 6 dpi. In contrast to the mock infection (PBS), A/CA04 infection resulted in the detection of both NP and pTrkA, which were mostly localized to lung epithelium (**Fig 1G**). Taken together, IAV infection strongly activates the TrkA signaling in the lungs.

### Inhibition of the TrkA signaling protects mice from lethal IAV infection

**Fig 2:**
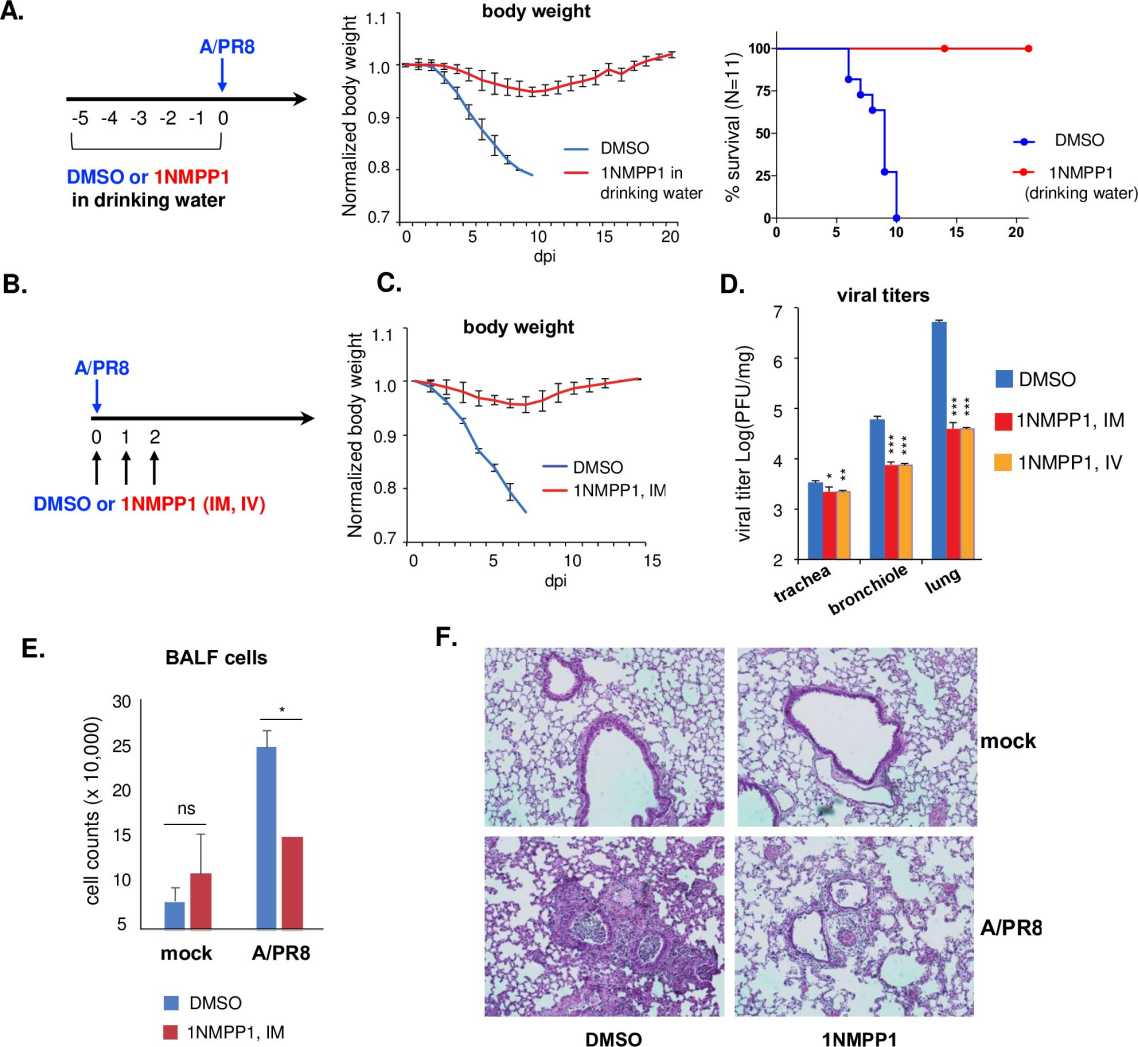
Inhibition of the TrkA signaling protected mice from lethal A/PR8 infection. **(A)** TrkA KI mice were given DMSO or 1NMPP1 in drinking water for 5 days before infection (IN) with 5x MLD_50_ of A/PR8 and monitored for body weight and survival. Normalized body weight was shown as average + SEM. Survival curves were compared by log-rank (Mantel-Cox) χ^2^ test. **(B)** TrkA KI mice were infected (IN) with 5x MLD_50_ of A/PR8 and treated with either DMSO or 1NMPP1 through IM or IV inoculation. The IV route was used only for the comparison of viral titers, whereas IM was used in all experiments. Normalized body weight was shown as average + SEM **(C),** Viral titers in trachea, bronchiole, and lungs at 3 dpi were measured by plaque assay **(D).** BALF cells at 6 dpi were counted **(E).** Lung pathology at 6 dpi was analyzed by H&E staining of the lung section **(F)**. A representative image from 1NMPP1 treatment via IM route was shown. Statistical analysis of viral titer and BALF cell count was conducted by Student’s t-test. ns, not statistically significant. *, p < 0.05. **, p<0.01. ***, p<0.001.

We used the TrkA^F592A^ KI mouse strain to specifically turn off the TrkA kinase activity through the use of a small molecule 1NMPP1 [36]. We first delivered 1NMPP1 or vehicle control DMSO in drinking water for 5 days, which was applied in previous studies [36] and did not cause any noticeable adverse effects on mice, and then challenged mice with a lethal dose of the mouse-adapted A/PR8 strain at 5xMLD50. Mice in the DMSO control group all succumbed to the infection by day 10, while mice in the 1NMPP1 group all survived the infection with slight body weight loss from day 4 to 10 (**Fig 2A**).

To quantitatively control the 1NMPP1 dosage that each mouse receives, we decided to give 1NMPP1 through IM or IV route (**Fig 2B**). The IV route was tested in a relatively small number of mice for the sole purpose of a comparison of viral titers in trachea, bronchiole, and lungs at 5 dpi to the IM route. In the DMSO control group, A/PR8 was detected throughout the respiratory system, at ~ 8x10^6^ PFU/mg in lungs, ~ 8x10^5^ PFU/mg in bronchiole, and ~ 5x10^3^ PFU/mg in trachea. Administration of 1NMPP1 through either the IM or IV route significantly reduced viral loads in all respiratory tissues, in particular, in the lungs by about 2 logs (**Fig 2D**), demonstrating that TrkA inhibition reduced influenza viral replication *in vivo*. As no significant difference in viral titer was detected between the IM and IV routes of 1NMPP1 delivery, subsequent experiments used the IM route only. 1NMPP1-treated mice all survived the lethal A/PR8 infection with only a slight body weight loss (**Fig 2C**). Virus-induced lung inflammation was evaluated by bronchial alveolar lavage fluid (BALF) cell counts and lung histopathology analysis. A/PR8 infection increased the number of BALF cells that were significantly reduced by 1NMPP1 treatment (**Fig 2E**), suggesting that TrkA inhibition could reduce the degree of virus-induced lung inflammation. This finding was validated by the histopathological analysis of the lung sections (**Fig 2F**). Compared to mock-infection control, A/PR8 infection resulted in significantly increased infiltration of inflammatory cells, damaged epithelium, and cell debris in bronchiolar lumen. 1NMPP1 inoculation alone did not cause lung tissue damage or inflammation but significantly reduced the degrees of airway epithelial cell damage and inflammatory cell infiltration in A/PR8-infected lungs. Taken together, using a chemical genetic approach to specifically inhibit the TrkA activation in mice, we have shown that the TrkA signaling plays an important role in the IAV infection *in vivo*.

### Cell-type-specific role of TrkA activation in the IAV replication

**Fig 3:**
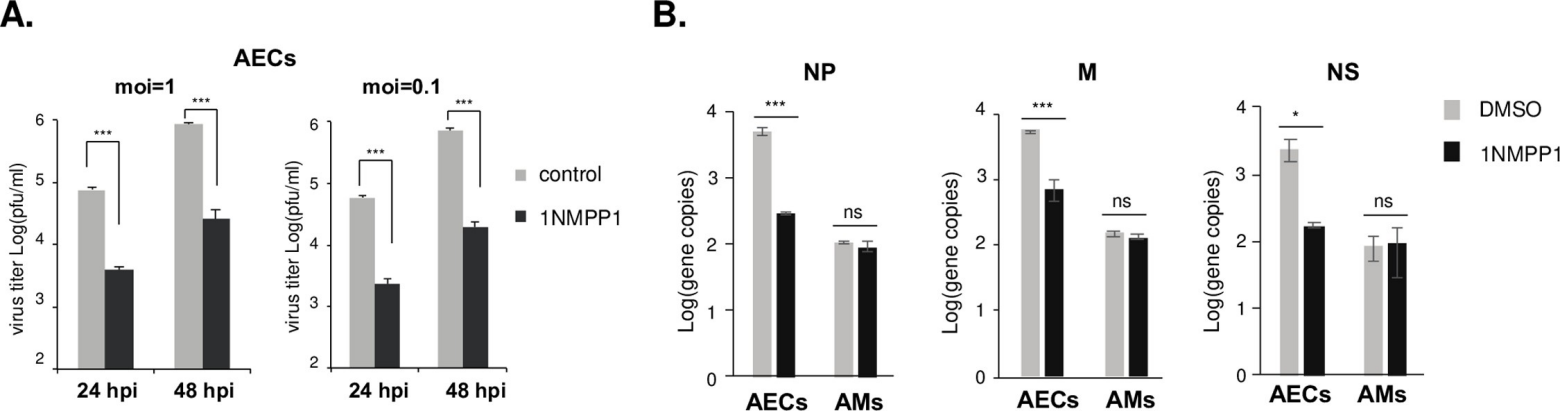
TrkA signaling is important for IAV replication in mouse AECs but not in AMs. Primary AECs and AMs isolated from TrkA KI mice were infected with A/WSN at MOI of 0.1 or 1, in the presence of either DMSO or 1NMPP1. **(A)** Viral titers in the supernatants from AECs at 24 and 48 hpi were quantified by plaque assay. **(B)** Viral genes NP, M, and NS in the infected AECs and AMs were quantified by qRT-PCR. Statistical analysis was conducted by Student’s t-test. ns, not statistically significant. *, p < 0.05. **, p<0.01. ***, p<0.001.

To determine the specific role of the activated TrkA signaling in different cell types, we isolated primary AECs and AMs from the TrkA KI mice, and infected them with A/WSN at MOI of 1 and 0.1, respectively, in the presence of either DMSO or 1NMPP1. Viral titers in the supernatants at 24 and 48 hpi were quantified by plaque assay. Consistent with our previous findings [10,11], 1NMPP1-mediated specific TrkA inhibition led to significantly reduced viral titers by ~ 2 logs at both time points and with both MOIs in AECs (**Fig 3A**), demonstrating the important role of TrkA in the influenza viral replication in respiratory epithelial cells. As AMs do not support productive viral replication of various IAV strains [39–42], we did not detect any infectious virus in the supernatants from A/WSN-infected AMs, with or without TrkA inhibition.

**Fig 4:**
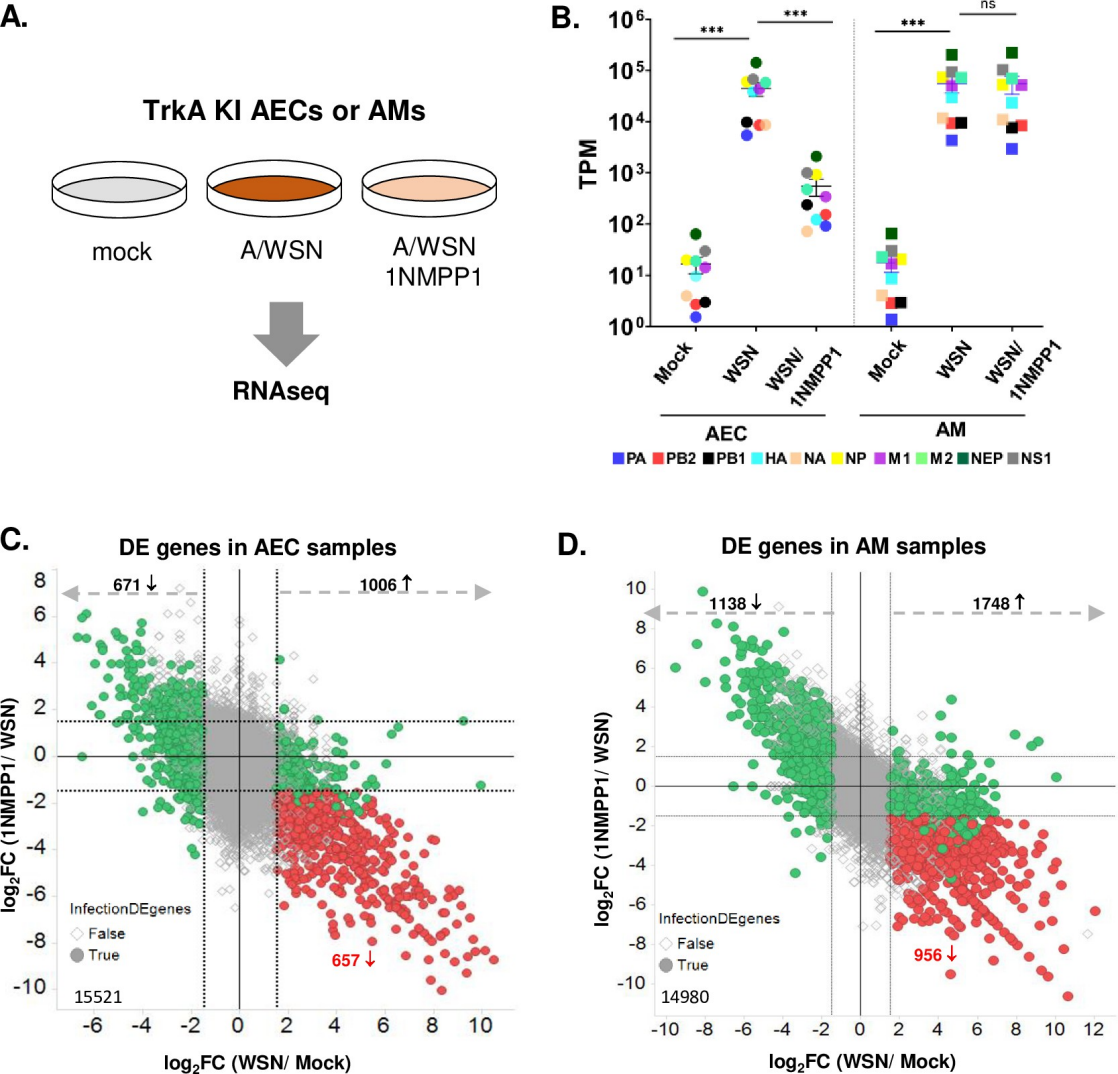
RNAseq analysis of IAV-infected AECs and AMs with and without TrkA inhibition. **(A)** Primary AECs and AMs from TrkA KI mice were mock infected, infected with A/WSN at MOI of 10 with or without 1NMPP1, each in triplicates. Total RNAs from each condition were submitted for RNAseq analysis. **(B)** Transcripts mapped to A/WSN viral genes are shown as TPM for each condition in AECs and AMs. Statistical analysis was conducted by Student’s t-test. ns, not statistically significant. ***, p<0.001. Error bar = SEM. The differentially expressed (DE) host genes from pair comparisons in AECs **(C)** and AMs **(D)**. Genes that were differential expressed by WSN infection (•, green and red) were identified by having absolute log2FC(WSN/Mock) > = 1.5. TrkA-regulated genes (red) were selected from the pool of infection’s DE genes if the effect from infection (up-regulated or down-regulated) was reversed at least 1.5-fold. Total number of transcripts included in the analysis was shown in lower left (15521 –AEC, 14980 –AM).

We further compared the level of viral RNA synthesis in AECs and AMs with or without TrkA inhibition by qRT-PCR using viral gene-specific primers (**Fig 3B**). For all three viral genes (NP, M1, and NS) tested, TrkA inhibition by 1NMPP1 led to a significant reduction of viral RNAs by 1–1.5 logs in AECs but had no effect on viral RNA levels in AMs (**Fig 4B**). The results suggest that the TrkA signaling is important for IAV replication, especially viral RNA synthesis, in epithelial cells but not in macrophages.

### Transcriptomic analysis of IAV-infected AECs and AMs

To further characterize the cell-type-specific role of the TrkA signaling in IAV infection, we conducted transcriptomic analysis of virus-infected AECs and AMs with or without the TrkA inhibition. Primary AECs and AMs isolated from TrkA KI mice were mock infected, infected with A/WSN, or infected with A/AWSN in the presence of 1NMPP1, each in triplicates (**Fig 4A**). Total RNAs at 10 hpi were analyzed by RNA-sequencing. After stringent data filtering, sequencing reads were mapped to the combined mouse and A/WSN viral genomes (mouse+IAV) and the TPM (transcript per million) values were calculated for all transcripts. TPM distribution and MDS (Multi-Dimensional Scaling) Plot were used to assess data correlation. While all AEC samples showed distinct clusters of gene expressions for different treatments and almost identical TPM distribution profiles between replicates, two AM samples exhibited non-uniform TPM distribution and were not clustered well with other within-group replicates (**S1 Fig**). These two samples, one from the mock infection and the other from IAV infection, could be outliers due to technical issues during the sample preparation steps, and were excluded from differential expression analysis.

### Analysis of viral RNA levels in AECs and AMs by RNA-seq analysis

For both AECs and AMs, sequencing reads from mock-infected cells were nearly all mapped to mouse genome, whereas those from A/WSN-infected cells were 59%-75% mapped to mouse genome and 25%-41% mapped to the viral genome **(S2 Fig**), which confirmed the efficient viral RNA synthesis in both cell types after viral infection. 1NMPP1 treatment almost completely reduced the percentage of viral genome reads in AECs but did not alter that in AMs (**S2A Fig**). Similar observations were made with the percentage of transcripts (**S2B Fig**), consistent with the findings that TrkA inhibition effectively reduced IAV RNA synthesis in AECs but not in AMs (**Fig 3**).

We also used TPM to evaluate the levels of viral transcripts and plotted the average TPM for each of the 10 viral transcripts (PB2, PB1, PA, HA, M1, M2, NP, NS1, NEP, NA) under three experimental conditions (mock, A/WSN, and A/WSN with 1NMPP1) for AECs and AMs, respectively (**Fig 4B**). All viral transcripts were below 10^2^ TPM in the mock-infected cells and reached 10^4^−10^5^ TPM in both AECs and AMs after viral infection. 1NMPP1-mediated TrkA inhibition significantly reduced the level of viral transcripts by ~ 2 logs TPM in AECs but did not affect those in AMs (**Fig 4B**). Thus, in consistent with the qRT-PCR data (**Fig 3**), the RNAseq results support the cell-type-specific role of the TrkA signaling in promoting IAV replication in epithelial cells but not in macrophages.

### TrkA-regulated host gene expression in IAV-infected AECs and AMs

Differential gene expression was performed between two experimental conditions, with the cutoff as the absolute log2 fold change (Log2FC) larger than 1.5 and FDR smaller than 0.05 (**S3A and S3B Fig**). The upregulated and downregulated genes in each pair-wise comparison are shown in dot plots for AECs (**Fig 4C**) and AMs (**Fig 4D**). The number of differentially expressed (DE) genes also shown in Venn diagram (**S3C and S3D Fig**). Overall, A/WSN infection led to 1006 increased transcripts and 671 decreased ones in AECs, 1748 increased transcripts and 1138 decreased ones in AMs. 1NMPP1-mediated TrkA inhibition reversed many but not all of the virus-altered genes, suggesting that some genes are under the direct and/or indirect regulation of the TrkA signaling while others are not.

We are particularly interested in genes that are upregulated by A/WSN infection through TrkA signaling, as they may play important roles in viral replication and disease pathogenesis. The flowchart to identify TrkA-regulated genes in virus-infected cells is shown in **S4 Fig**. Among the 1006 A/WSN-activated genes in AECs, 657 genes were reduced by 1NMPP1 treatment. Among the 1748 A/WSN-activated genes in AMs, 956 genes were reduced by 1NMPP1 treatment. Thus 657 AEC genes and 956 AM genes were upregulated via the TrkA activation in A/WSN-infected cells. Only 113 genes (11–14%) were common in both cell types, suggesting that IAV-induced TrkA activation results in mainly cell-type-specific gene expression patterns in AECs and AMs.

**Fig 5:**
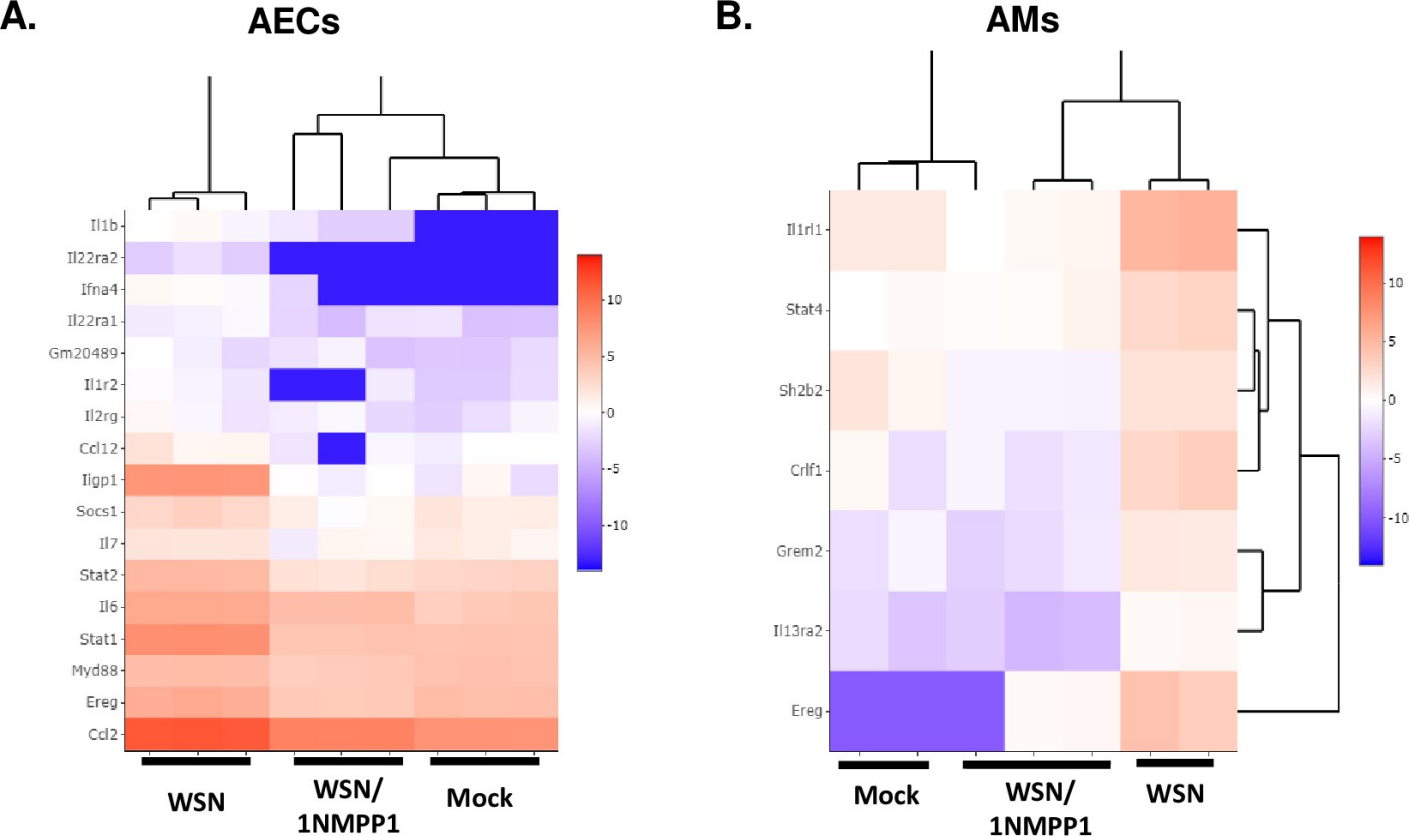
TrkA signaling regulates the expression of pro-inflammatory genes in IAV-infected AECs and AMs. TrkA-regulated inflammatory genes in AEC **(A)** and AM **(B)** are shown in heatmaps.

Functional enrichment analysis of the 657 TrkA-upregulated AEC genes (**S1 List**) showed that they were mostly involved in the cell defense response to viruses, such as the interferons (IFNs) and IFN-stimulated genes (ISGs). Among the targets are genes regulating the inflammatory responses such as cytokine/chemokine production and signaling, IL-1β production, and lymphocyte chemotaxis. For example, IL-6, IL-1β, and IFNs are known mediators of cytokine storms. As shown in the heatmap (**Fig 5A**), their transcript TPMs were increased in virus-infected cells and reduced in virus-infected 1NMPP1-treated cells. These results suggest that TrkA might serve as a major activator of inflammatory responses in A/WSN-infected AECs.

In A/WSN-infected AMs, TrkA was found to mainly regulate the complement activation, B cell receptor signaling, phagocytosis, and IFN-I signaling (**S2 List**). TrkA also regulated a small subset of proinflammatory genes in AMs, such as CCL5, CCL11, CXCL5, CXCL14, IL19, IL23, IL24, IL33, and IL1RL1. Their transcript TPMs were shown in the heatmap (**Fig 5B)**. As TrkA inhibition did not affect viral RNA synthesis in AMs, this result suggests that TrkA could promote an inflammatory response in AMs independent of its role in IAV replication.

### Inhibition of the TrkA signaling reduced IAV-induced airway inflammation *in vivo*

**Fig 6:**
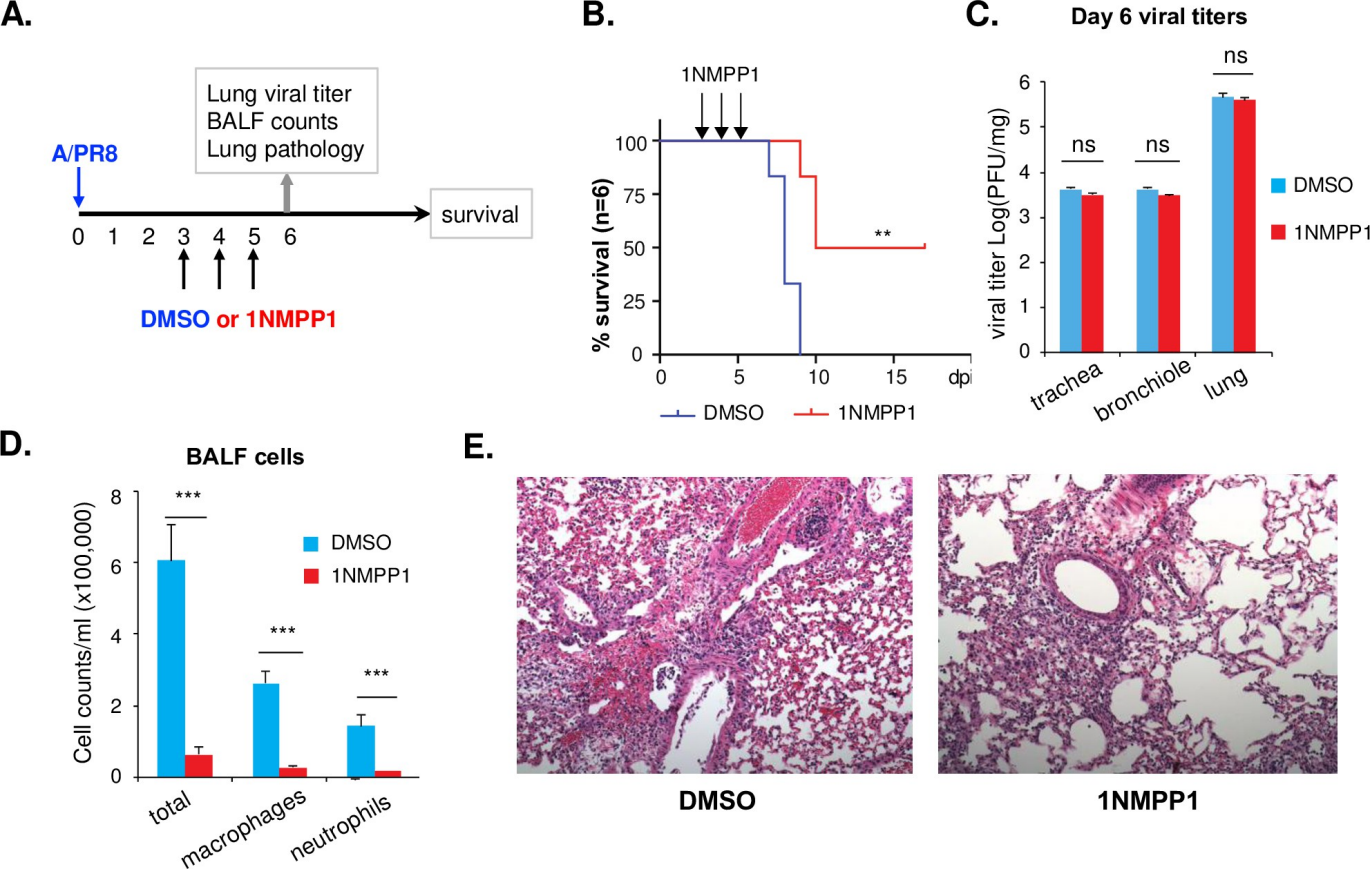
Inhibition of the TrkA signaling reduces the A/PR8-induced airway inflammation. **(A)** TrkA KI mice were infected (IN) with A/PR8 of 5xMLD50 and treated (IM) with either DMSO or 1NMPP1 on day 3, 4, and 5. **(B)** Survival curves were compared by log-rank (Mantel-Cox) χ^2^ test. **(C)** Viral titers in trachea, bronchiole, and lungs at 6 dpi were measured by plaque assay. **(C)** Total cells, macrophages, and neutrophils from BALF at 6 dpi were counted. **(E)** Lung pathology at 6 dpi were analyzed by H&E staining of the lung section. Statistical analysis of viral titer and BALF cell count was conducted by Student’s t-test. ns, not statistically significant. *, p < 0.05. **, p<0.01. ***, p<0.001.

The RNA-seq results suggest that the TrkA signaling might play an important role in virus-induced airway inflammation by activating the inflammatory responses in viral target cells. To determine whether TrkA can directly promote the airway inflammation independent of its role in viral replication, we asked whether applying the TrkA inhibitor after the peak of viral replication would be able to reduce the airway inflammation without affecting viral titer. A/PR8 viral titer peaks in the mouse lungs at 3 dpi, while airway inflammation and lung pathology continue to develop afterwards. We thus treated A/PR8-infected TrkA KI mice at 3 dpi with either vehicle control DMSO or 1NMPP1 (IM) for 3 days (**Fig 6A**). All mice in the control group succumbed to the A/PR8 infection by 9 dpi, whereas 50% of mice in the 1NMPP1 treatment group survived the infection with a transient body weight loss (**Fig 6B**). As expected, addition of 1NMPP1 from 3–5 dpi did not affect viral titers, as shown by the similar viral loads in trachea, bronchiole, and lung at 6 dpi between the DMSO and 1NMPP1 groups (**Fig 6C**). In contrast, significantly lower BALF cell counts, both total and specific cells (macrophages and neutrophils), were observed in the 1NMPP1 group than in the DMSO group (**Fig 6D**). Consistently, less lung pathology and less inflammatory cell infiltration were found in the 1NMPP1 group than in the DMSO group (**Fig 6E**). Similar experiment was also conducted in TrkA KI mice infected with H1N1pdm strain A/CA04 (**S5 Fig**). 1NMPP1 treatment applied at 3–5 dpi did not affect viral load in the lungs (**S5B Fig**) but reduced the BALF cell count (**S5C Fig**) and lung inflammation (**S5D Fig**). Taken together, these data suggest that 1NMPP1-mediated specific TrkA inhibition can reduce the IAV-induced lung inflammation independent of its direct antiviral activity, supporting a direct role of the TrkA signaling in the IAV-induced airway inflammation.

## Discussion

Influenza virus-induced respiratory disease is due to not only the direct cytopathic effect of viral infections but also the exacerbated inflammatory host responses. Effective therapeutics should therefore reduce both viral replication and pathogenic airway inflammation. Using TrkA KI mice and the derived primary pulmonary cells, in which the TrkA receptor tyrosine kinase activity can be specifically and effectively turned off by a small molecule 1NMPP1, we show in this study that the TrkA signaling is an important pathway to mediate both IAV replication and virus-induced airway inflammation, and that targeting TrkA signaling may provide an effective measure to protect against respiratory influenza disease.

**Fig 7:**
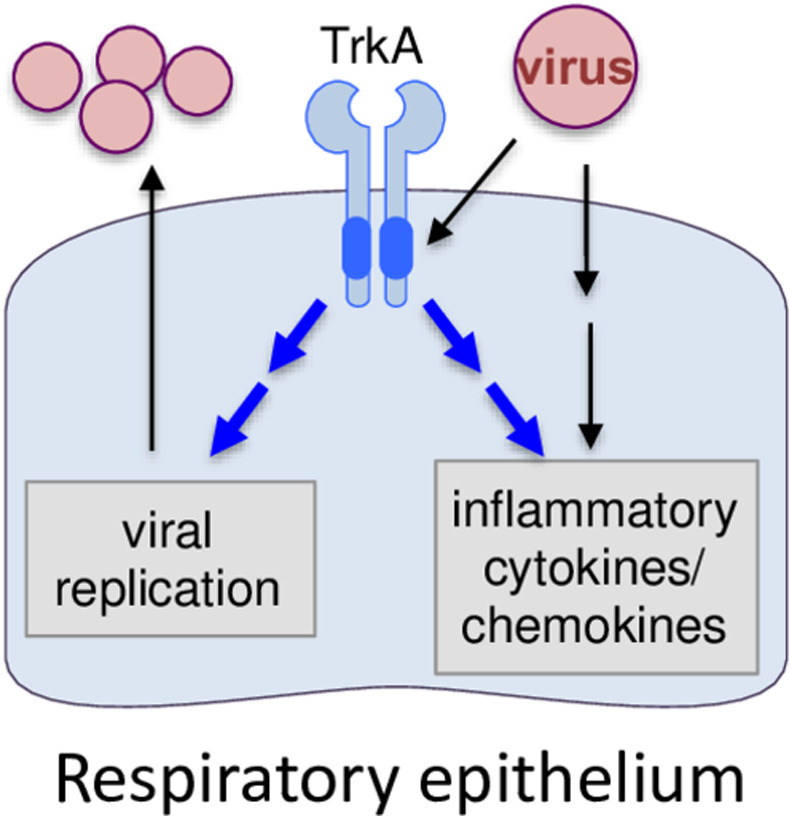
A proposed model on the role of TrkA signaling in the influenza viral replication and virus-induced airway inflammation. Influenza viral infection activates TrkA signaling, which in turn activates distinct target genes in different cell types that function to increase viral replication in AECs and to promote inflammatory responses in both AECs and AMs.

TrkA is widely expressed in a variety of cell types in the lungs, including AECs and AMs [23,24] that are known target cells of IAV infection. Using Western blotting and IFA, we show that IAV can readily activate TrkA signaling in both AECs and AMs early after viral infection and in the lungs of IAV-infected mice (**Fig 1**). To evaluate the specific role of TrkA in IAV infection *in vivo*, we administered 1NMPP1, the specific TrkA inhibitor in TrkA^F592A^ KI mice, through drinking water or through IM or IV route, and demonstrated its full protection against a lethal IAV infection, with a significantly reduced viral titer in the respiratory systems, reduced airway inflammation and lung pathology (**Fig 2**). Consistently, 1NMPP1 significantly reduced viral replication and viral RNA synthesis in the primary AECs from TrkA KI mice, but surprisingly did not affect the viral RNA replication in primary AMs, suggesting a cell-type-specific role of TrkA in IAV replication (**Fig 3**). Transcriptomic analysis of virus-infected AECs and AMs, with or without TrkA inhibition, not only confirmed the cell-types-specific functional role of TrkA in viral RNA synthesis in AECs (**Fig 4B**), but also revealed TrkA-regulated genes in both cell types. As proinflammatory genes are under the TrkA regulation in virus-infected AECs and AMs, we tested the hypothesis that TrkA activation can directly upregulate pro-inflammatory genes to promote inflammation, by applying 1NMPP1 to virus-infected TrkA KI mice at 3 dpi when virus replication had already peaked in the lungs. Indeed, TrkA inhibition at 3 dpi did not affect viral load in the lungs (6 dpi) but caused a significant reduction in the level of lung inflammation and provided partial protection against a lethal IAV infection (**Fig 6**). Taken together, these data suggest that IAV infection of pulmonary cells can activate the TrkA signaling, which functions to increase viral RNA synthesis and viral replication in AECs, and to promote inflammatory responses by activating distinct sets of pro-inflammatory genes in both AECs and AMs (**Fig 7**).

**TrkA signaling promotes the influenza viral replication in AECs.** We have previously shown that TrkA signaling is important for influenza A and B viral replication [10,11] and that it is involved in multiple steps of viral life cycle including viral RNA synthesis [11]. In this study, we have confirmed the functional role of TrkA signaling in the influenza viral replication and further demonstrated its cell type specificity. Both RT-qPCR (**Fig 3B)** and RNA-seq (**Fig 4B**) results conclude that TrkA signaling is important for viral RNA synthesis in AECs but not in AMs. The underlying mechanism is unknown and may involve AEC-specific TrkA-regulated host factor(s) that are important for influenza viral RNA synthesis. TrkA regulates a distinct pattern of genes in AECs and in AMs after viral infection, with only 11%-14% shared targets (**S4 Fig**), suggesting that TrkA signaling has differential functions depending on the cellular context. Further studies are required to fully understand its functional mechanism in mediating influenza viral replication in AECs.

### TrkA signaling activates inflammatory responses in IAV-infected cells

Transcriptomic analysis identified pro-inflammatory genes as TrkA-activated targets in IAV-infected AECs and AMs (**Fig 5**), including IL-6, IL-1β, IFNα, CCL2, CCL5, CXCL-9, CXCL-10, IL33, and IL1RL1/ST2. Many of these cytokines/chemokines are highly elevated in the cytokine storm underlying the pulmonary inflammation in severe influenza diseases [43]. In particular, IL-6 is the key cytokine underlying the cytokine release syndrome, a systemic inflammatory response triggered by various factors including viral infections such as severe influenza and COVID-19 [44–46]. IL-33 binding to its receptor IL1RL1/ST2 is known to activate many pro-inflammatory cytokines and has been shown to mediate inflammation in respiratory diseases [47–49]. Our study suggests that the TrkA signaling can directly and indirectly induce the production of major inflammatory cytokines/chemokines from IAV-infected cells, causing airway inflammation and lung pathology. Thus, targeting TrkA to mitigate virus-induced hyperinflammation can be a potential therapeutic to treat severe influenza disease. Indeed, TrkA inhibitor administered after the peak of IAV replication did not reduce viral load in the lungs but was able to decrease lung inflammation and provide partial protection in mice (**Figs 6** and **S5**). Together with the accumulating evidence for a pathophysiological role of TrkA signaling in allergic airway inflammation [25–30], our study implicates TrkA as a common signaling pathway mediating pulmonary inflammation induced by both viral and non-viral factors.

In summary, we have shown that IAV infection activates TrkA signaling, which in turn activates distinct target genes in different cell types that function to increase viral replication in AECs and to promote inflammatory responses in both AECs and AMs (**Fig 7**). Our study suggests TrkA as an attractive host target for developing effective therapeutics against influenza viral diseases by blocking both viral replication and airway inflammation.

## Materials and methods

### Ethics statement

Research conducted for this manuscript was approved by the Institutional Biosafety Committee at the University of Minnesota, Twin Cities, under the protocol ID 2008-38359H. All animal procedures were approved by the Institutional Animal Care and Use Committee (IACUC) of University of Minnesota, Twin cities, under the protocol ID 2006-38173A.

### Cells, viruses, antibodies, and compounds

A549 cells (human lung epithelial cells) were grown in Dulbecco’s modified Eagle’s medium (DMEM) supplemented with 10% heat-inactivated fetal bovine serum (FBS). Madin-Darby canine kidney (MDCK) cells were maintained in Eagle’s minimal essential medium (MEM) supplemented with 5% FBS. All chemical compounds were purchased from Sigma.

Influenza virus strains (A/WSN/33, A/PR8/34, A/California/04/2009 (A/CA04)) were grown in 10-day-old embryonated chicken eggs. The viral titers were determined by plaque assay on MDCK cells. After infection with influenza A virus, MDCK cells were grown in low-serum L-15 medium, which consists of 15 mM HEPES (pH 7.5), nonessential amino acids, 0.75 g of sodium bicarbonate per liter, and 0.125% (w/v) bovine serum albumin (BSA). With the exception of A/WSN/33, infection of cultured cells with influenza viruses was conducted in the presence of trypsin at a concentration of 2.5 μg per ml.

Anti-influenza NP mouse antibody (MCA400) was purchased from Bio-Rad (Hercules, CA). Anti-TrkA rabbit antibody (Cat#2505) and anti-phospho-TrkA (pTrkA) rabbit antibody (Cat #9141) were purchased from Cell Signaling Technology (Danvers, MA). Anti-GAPDH mouse monoclonal antibody (G8795) was purchased from Sigma-Aldrich (St. Louis, MO).

1NMPP1 (Axon 1892, Axon Medchem, Reston, VA) was dissolved in DMSO at the concentration of 50 mg/ml. On the day of use, 1NMPP1 was first diluted to 10 mg/ml in DMSO, and further diluted in PBS for the appropriate dosages. GW441756 (Sigma) was used at 10 μM in A549 cells.

### Influenza A virus infection of mice

A TrkA^F592A^ knockin (TrkA KI) mouse line [36] was obtained from Dr. Keqiang Ye at Emory University and maintained in the research animal resources facility of University of Minnesota. Six-to 12-week-old mice were anesthetized by isoflurane and infected intranasally with 5xMLD_50_ of A/PR8 or A/CA04 in 50 μl volume. Animals were given either DMSO/PBS (16% v/v) or 1NMPP1 at 10 mg/kg/day by intramuscular (IM) or intravenous (IV) injection. In one study 1NMPP1 was given to mice in drinking water at 25 μM. Mice were monitored daily for clinical signs and body-weight loss up to day 21. Mice were euthanized if they reached pre-specified terminal points as previously described [50]. Mice were euthanized at specified time points. Bronchoalveolar lavage fluid (BALF) was collected for total and differentiated cell count after staining with Hema 3 System (Thermo Fisher Scientific). Upper and lower respiratory tracts, and right lungs were collected and homogenized for viral quantification by plaque assay. Left lungs were first perfused with 4% paraformaldehyde (PFA), fixed in 4% PFA and paraffin embedded for histopathological analysis.

### Histopathological analysis and immunofluorescence assay (IFA)

As described previously [30], paraffin-embedded lung tissue sections (4 μm) were stained with H&E (Leica Biosystems Inc.) and viewed under a microscope. For IFA, lung tissue sections, after antigen retrieval, were first stained with anti-NP mouse antibody and anti-pTrkA rabbit antibody, followed by incubation with FITC-conjugated anti-mouse IgG and Rhodamine-conjugated anti-rabbit IgG secondary antibodies (Jackson ImmunoResearch Laboratories). Mouse and rabbit IgG antibodies were included as IgG controls. 4,6-diamidino-2-phenylindole (DAPI) was used to visualize nuclei and slides were examined by confocal microscopy (Olympus *Fluoview 1000)*.

### Isolation of primary AECs and AMs

Primary alveolar epithelial cells (AEC) and alveolar macrophages (AM) cells were isolated from TrkA KI mice following the established protocols [51,52]. After euthanization, mouse lungs were perfused with sterile HBSS (CX30300, Invitrogen), and filled with Dispase (CB40235, Fisher Scientific) followed by 1% low-melt agarose (CX25009, Bioexpress). Lungs were immersed in Dispase for 45 min at room temperature, treated with DNase (AM2238, Invitrogen), mechanically minced into small pieces, and filtered through cell strainers to prepare lung single cell suspension. Cells were incubated with biotinylated anti-CD45 (BD Pharmingen BDB553078) and anti-CD16/CD32 (BD Pharmingen BDB553143) antibody mixture, followed by magnetic separation after addition of BioMag Nuclease-Free Streptavidin particles (Qiagen, 311711). The enriched AEC suspensions were collected and cultured in DMEM/F12 Medium (Lonza, 12001–600) supplemented with 10% FBS on tissue culture-treated dishes.

AMs were harvested from euthanized mice by bronchoalveolar lavage. The pooled lavage fluid was centrifuged for 15 min at 1500 rpm. Cells were re-suspended in DMEM/F12 Medium (Lonza, 12001–600) supplemented with 10% FBS, counted in a hemocytometer, and plated to a cell culture dish for adhesion. The adherent cells were greater than 90% macrophages, as assessed by Diff-Quick staining.

### RNA-Sequencing and RNA-Seq data analysis

Primary AECs and AMs seeded on 6-well plates were subjected to three different treatments: (1) mock infection, (2) A/WSN infection (MOI = 10), and (3) A/WSN infection (MOI = 10) in the presence of 1NMPP1 (10 μm), each in triplicates. Total RNAs at 10 hpi were stored in RNAlater and submitted to University of Minnesota Genomic Center (UMGC) for RNA extraction, mRNA enrichment, library construction, and RNA-sequencing. The cDNA libraries of the mRNAs of all 18 samples were created using TruSeq RNA Library Prep Kit v2 (Illumina, San Diego, CA). All the libraries were sequenced on a HiSeq 2500 High Output using v4 chemistry platform to generate approximately 220 million 50-bp pair-end reads. The transcriptomic data are available in NCBI Gene Expression Omnibus (GEO) (accession no GSE203539). All the raw RNA-Seq reads were processed with adapter trimming and low-quality base removal by Trimmomatic [53] and checked for quality using FASTQC [54]. All trimmed reads with a minimum read length of 36bp and average quality per base higher than 30 were mapped into the combined genome (mouse+IAV) by STAR v2.5.3a [55].

Mouse reference genome, GRCm38, was downloaded from Ensembl. The IAV A/WSN genome was assembled by combining complete sequences for all eight genome segments from the Influenza Research Database [56] with NCBI Taxon ID NCBI:txid382835. The host genome and IAV genome were concatenated into one combined reference genome (Mouse+IAV). The annotation file was also curated by combining the annotation file for mouse (Mus_musculus.GRCm38.95.gtf) and IAV WSN.

All the alignment files were subjected to featureCounts [57] to quantify the number of reads mapped to gene features specified in the annotation file. The raw counts were then used to calculate TPM (Transcript Per Million) values for all transcripts. TPM distribution and MDS (Multi-Dimensional Scaling) Plot were used to assess data correlation.

EdgeR [58] was used to conduct differential expression analysis, in which a generalized linear model (GLM) was constructed for each cell type (AEC or AM) then quasi-likelihood F-test (QLF) was performed to determine differential expressed genes. Genes that have the absolute log2 fold change between two conditions larger than 1.5 and FDR smaller than 0.05 are determined to be significantly differential expressed.

Functional analysis was conducted using the Functional Annotation Tool of DAVID Bioinformatics Resources [59]. TrkA-regulated genes were used as input to identify associated biological terms from the database GOTERM_BP_DIRECT. Clusters that have enrichment scores higher than 1.3 were extracted and attached in S1 and S2 Lists. List of inflammatory chemokines and cytokines include the GO class GO:0019221 that was downloaded from ensemble using the package Biomart [60], as well as manually curated genes.

### Statistical analyses

Statistical analysis of the survival curve by log-rank (Mantel-Cox) χ^2^ test was conducted using GraphPad Prism 5 software. Statistical comparison of viral titers among different treatment groups throughout the manuscript was performed using Student’s t-test. *, P< 0.05, **, p<0.01, *** p<0.001.

## Supporting information

S1 Fig Assessment of RNA-Seq data profiles for AEC and AM samples. (A) MDS (Multi-Dimensional Scaling) Plot depicted the groups of RNA-Seq samples based on their similarity in RNA-Seq profiles (B) Distribution of TPM values in AEC samples (left) and AM samples (right). The outliers were framed in dotted circles (A) or plotted as dotted lines (B).
(TIF)

S2 Fig Distribution of reads mapped to IAV and mouse genomes. **(A)** Percentage of reads mapped into IAV (red) or host genome (black) **(B)** Percentage of transcripts mapped into IAV (red) or host genome (black).
(TIF)

S3 Fig Pair-wise comparison between samples. Number of up-regulated (red) and down-regulated (blue) genes determined by quasi-likelihood F-test in edgeR package when comparing between **(A)** AEC samples, **(B)** AM samples. The horizontal dotted lines define the log2FC of -1.5 (lower) and 1.5 (upper). The number of differentially expressed (DE) host genes from pair comparisons in AECs (C) and AMs (D) is shown by Venn Diagram. The number of non-DE genes across all comparisons is shown at the right bottom corner.
(TIF)

S4 Fig Flow chart of the differential gene expression analysis conducted to identify the TrkA-upregulated host genes in virus-infected AECs (left) and AMs (right). Virus-altered host genes were first identified between the mock and A/WSN-infected cells. Among the virus-upregulated genes, those decreased by 1NMPP1 treatment are under the direct and indirect regulation of the TrkA signaling. Upregulated genes: FDR < 0.05 and Log2FC > 1.5. Downregulated genes: FDR < 0.05 and Log2FC < -1.5.
(TIF)

S5 Fig Inhibition of the TrkA signaling reduces the A/CA04-induced airway inflammation. **(A)** TrkA KI mice were infected (i.n.) with 1000 PFU of A/CA04 and treated (i.m.) with either DMSO or 1NMPP1 on day 3, 4, and 5. Animals were euthanized on day 5. Viral titers in the lungs were measured by plaque assay **(B)**. Total cells, macrophages, lymphocytes and neutrophils from BALF were counted **(C)**. Lung pathology at 6 dpi were analyzed by H&E staining of the lung section **(D)**. Statistical analysis of viral titer and BALF cell count was conducted by Student’s t-test. Ns, not statistically significant. *, p < 0.05. **, p<0.01.
(TIF)

S1 List Functional enrichment analysis of the TrkA-upregulated AEC genes. (XLSX)

S2 List Functional enrichment analysis of the TrkA-upregulated AM genes. (XLSX)
