# Supplementary figures and images for: Influenza A virus activates cellular Tropomyosin receptor kinase A (TrkA) signaling to promote viral replication and lung inflammation
Source: PLoS Pathog. 2022 Sep 19;18(9):e1010874. doi: 10.1371/journal.ppat.1010874 (PMC9521937; doi:10.1371/journal.ppat.1010874)

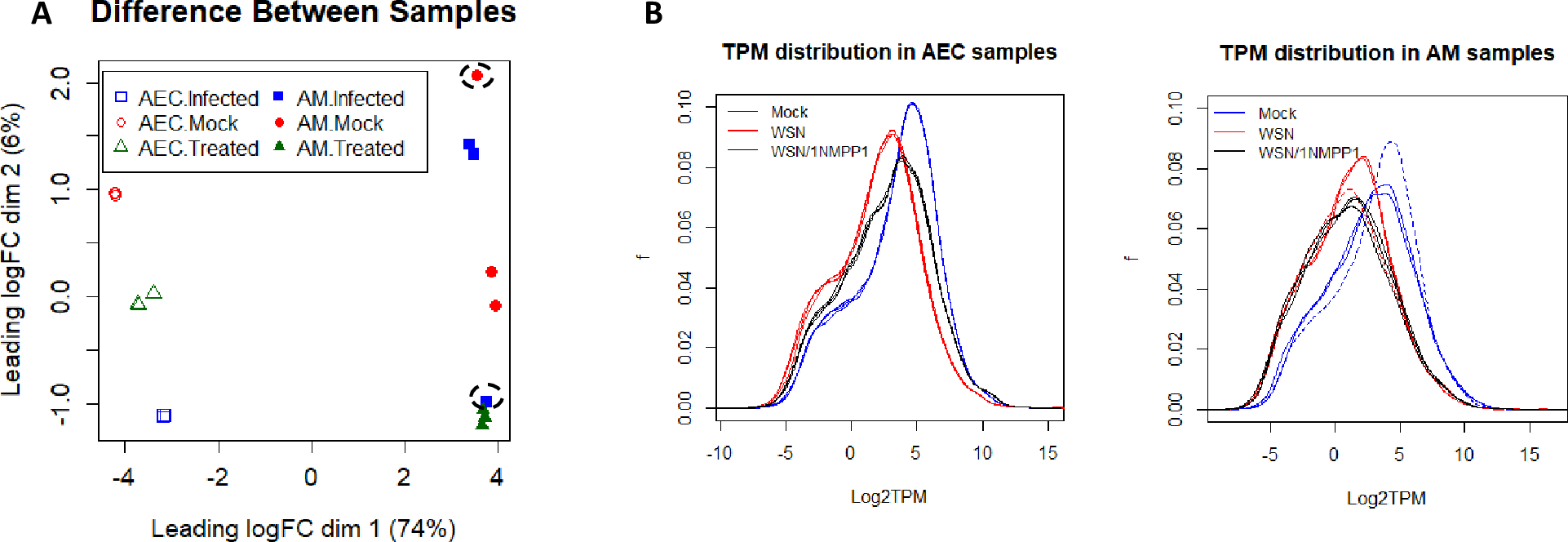

Supplement: S1 Fig — (A) MDS (Multi-Dimensional Scaling) Plot depicted the groups of RNA-Seq samples based on their similarity in RNA-Seq profiles (B) Distribution of TPM values in AEC samples (left) and AM samples (right). The outliers were framed in dotted circles (A) or plotted as dotted lines (B). (TIF) [file ppat.1010874.s001.tif]

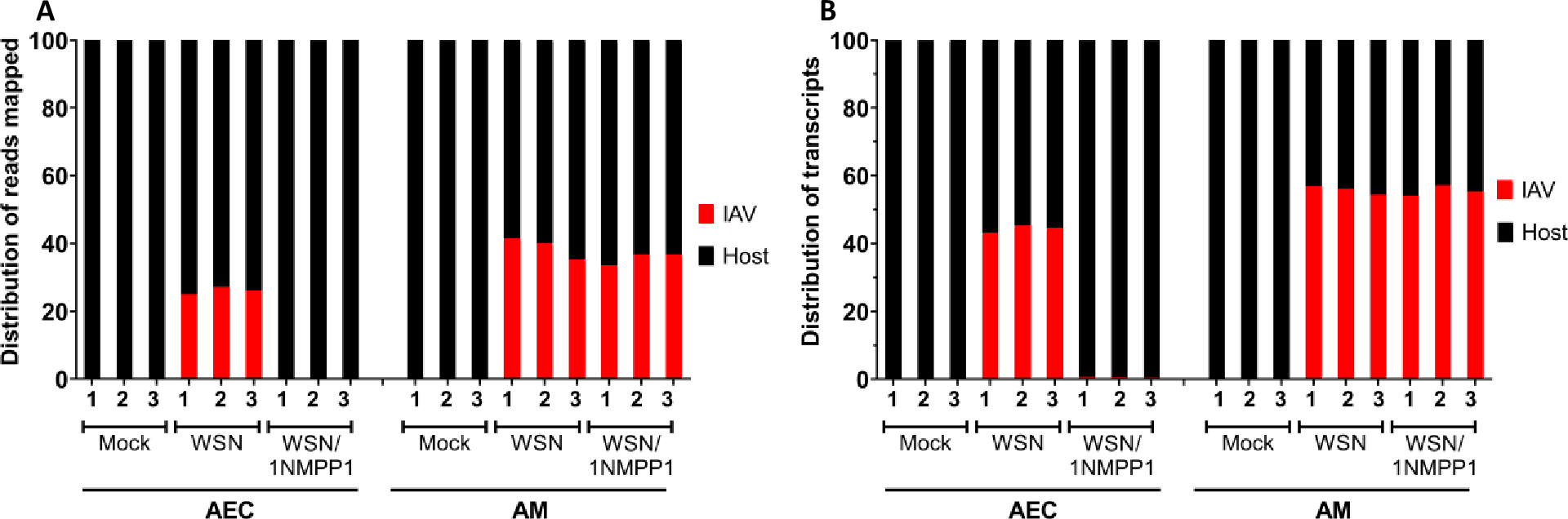

Supplement: S2 Fig — (A) Percentage of reads mapped into IAV (red) or host genome (black) (B) Percentage of transcripts mapped into IAV (red) or host genome (black). (TIF) [file ppat.1010874.s002.tif]

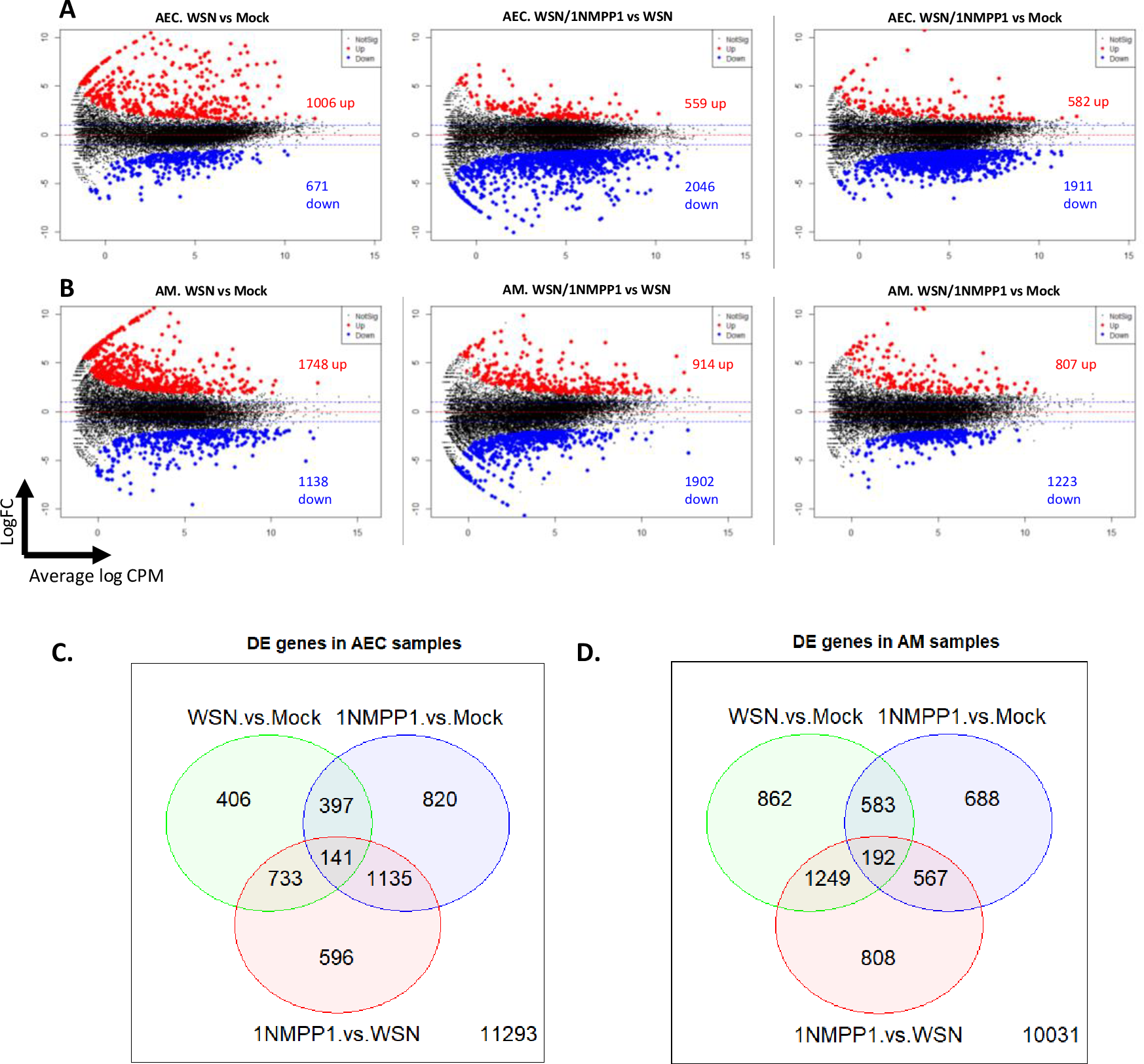

Supplement: S3 Fig — Number of up-regulated (red) and down-regulated (blue) genes determined by quasi-likelihood F-test in edgeR package when comparing between (A) AEC samples, (B) AM samples. The horizontal dotted lines define the log2FC of -1.5 (lower) and 1.5 (upper). The number of differentially expressed (DE) host genes from pair comparisons in AECs (C) and AMs (D) is shown by Venn Diagram. The number of non-DE genes across all comparisons is shown at the right bottom corner. (TIF) [file ppat.1010874.s003.tif]

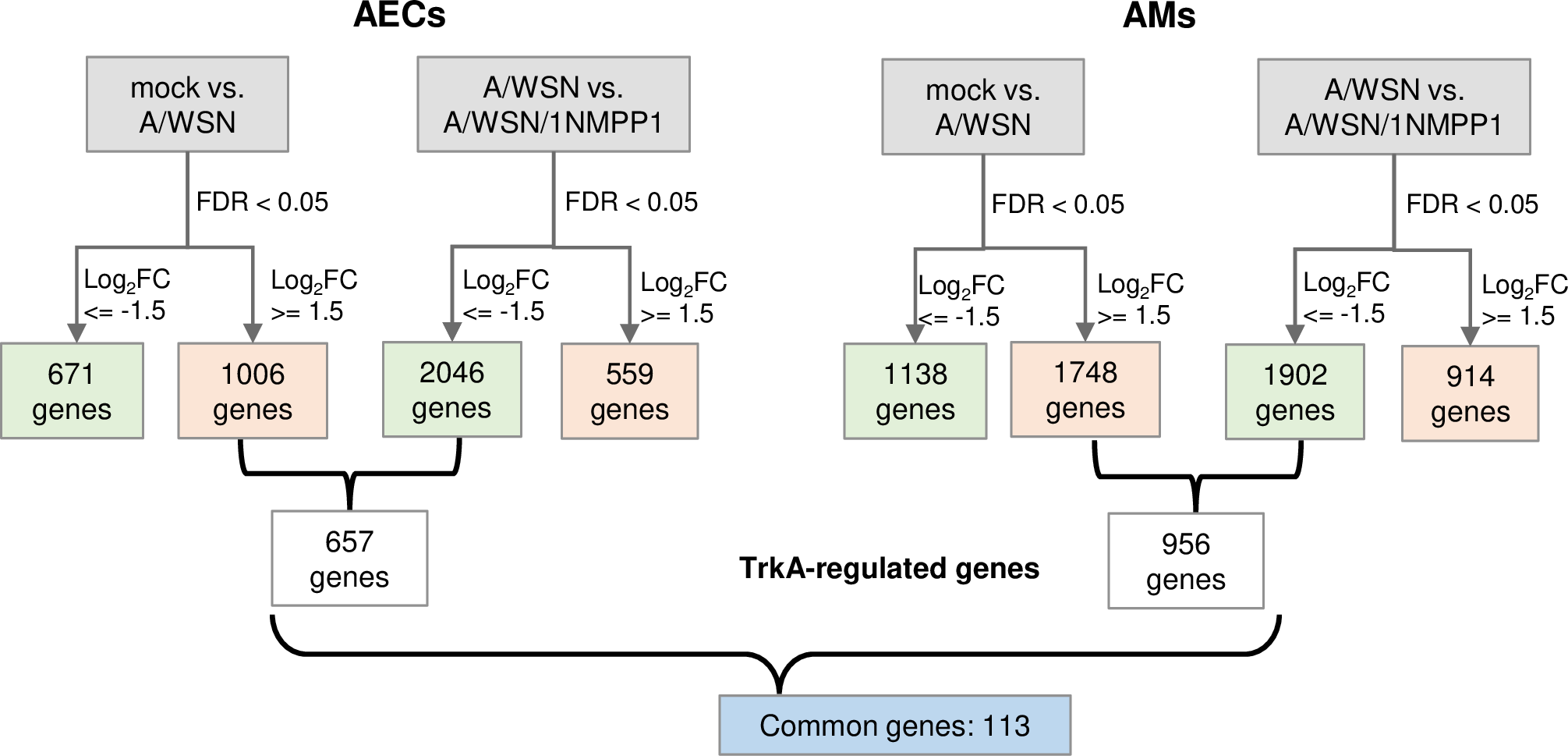

Supplement: S4 Fig — Virus-altered host genes were first identified between the mock and A/WSN-infected cells. Among the virus-upregulated genes, those decreased by 1NMPP1 treatment are under the direct and indirect regulation of the TrkA signaling. Upregulated genes: FDR < 0.05 and Log2FC > 1.5. Downregulated genes: FDR < 0.05 and Log2FC < -1.5. (TIF) [file ppat.1010874.s004.tif]

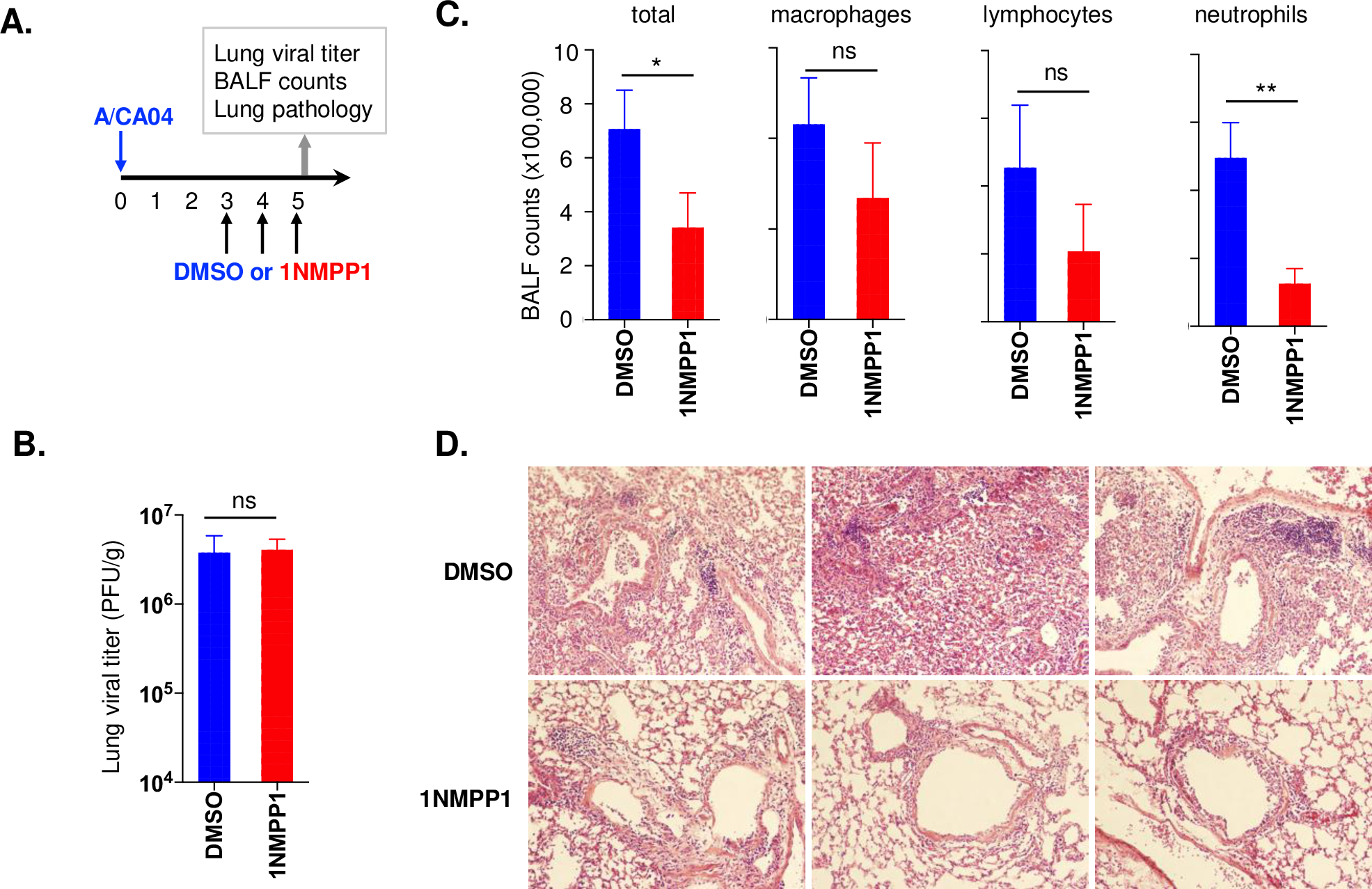

Supplement: S5 Fig — (A) TrkA KI mice were infected (i.n.) with 1000 PFU of A/CA04 and treated (i.m.) with either DMSO or 1NMPP1 on day 3, 4, and 5. Animals were euthanized on day 5. Viral titers in the lungs were measured by plaque assay (B). Total cells, macrophages, lymphocytes and neutrophils from BALF were counted (C). Lung pathology at 6 dpi were analyzed by H&E staining of the lung section (D). Statistical analysis of viral titer and BALF cell count was conducted by Student’s t-test. Ns, not statistically significant. *, p < 0.05. **, p<0.01. (TIF) [file ppat.1010874.s005.tif]
